# Indo-Pacific Order and Japan–India Relations in the Midst of COVID-19

**DOI:** 10.1177/2631684620940476

**Published:** 2020-09

**Authors:** Takenori Horimoto

**Affiliations:** 1 Gifu Women's University, Gifu, Japan.

**Keywords:** Covid-19, Indo-Pacific, Japan, India, multilateralism, regional order

## Abstract

Because of the USA’s relative decline of national power and the rapid emergence of China, the Indo-Pacific lacks a regional order as existed during the latter half of the twentieth century. The USA and China have had strained relations since the 2010s as economic and hegemonic rivals. Furthermore, at the cusp of the 2020s, a blame game is unfolding over COVID-19. Neither nation can be expected to serve a role as an order manager of peace and stability in the Indo-Pacific. Under such international situations, Japan and India should cooperate to initiate consideration of the regional order now. To establish such an order for the future, ends and means carry an importance. The ends should be the creation of a free, open, inclusive and democratic Indo-Pacific. The means should be some mechanisms based on principles of multilateralism, for example, Quad-Plus, not only involving the four countries: like-minded countries should also be included. In this way, we can find a silver lining beyond COVID-19.

**JEL Codes:** F5, I19

## Introduction

The Indo-Pacific as a region has been prevalently used since the 2010s. The twenty-first century was said to be the century of Asia. But this has to be renamed the century of the Indo-Pacific. The countries encompassing the Indo-Pacific are the USA, China, Japan, India, South Korea and Australia, to say the least of the Association of Southeast Asian Nations (ASEAN) and other littoral countries.

What are the main concerns of these countries over the Indo-Pacific? Perhaps, the USA as the major global power, albeit showing signs of relatively declining, might be concerned with the rise of an assertive China. China is yet to reach US position globally and might take more time. Therefore, it concentrates its primary focus on the Indo-Pacific, whereas the USA has no intention to yield any wiggle room to China and tries to prevent China from expanding its influence beyond the Indo-Pacific, more exactly East of Hawaii. The USA and China have had strained relations since the 2010s as strategic rivals. Now, at the cusp of the 2020s, a blame game is unfolding between the two over COVID-19.

When we delve into their rivalry, the cardinal aspect would be concerning the rivalry of regional hegemony. In other words, the essence of the rivalry might be boiled down to the tussle over the regional order in the Indo-Pacific. The order is yet to settle down in the twenty-first century, unlike the latter half of the twentieth century when the United States wielded the global hegemonic power, including over Asia.

Because the area of international politics has been expanded from the Asia-Pacific to the Indo-Pacific in this century, competing countries do not remain only the USA and China but other countries as mentioned above emerge as a major factor in formulating the regional order. They have their own strategic calculations and agendas to tide over the present power configurations in the Indo-Pacific.

Keeping such an overall picture in mind, this article delves into such aspects as regional order, order-building capability, competition among the major players like the USA, China, Japan, India and other important powers and a future perspective of the Indo-Pacific order.

## The Tussle Between the USA and China Over the Indo-Pacific Regional Order

When we look back at the global history, it would be axiomatic that a major power or a great power tends to build a more-or-less stable international order during the height of its power and influence, such as *Pax Romana, Pax Britannica*, and *Pax Americana,* for its own sake.

How about such an order in the twenty-first century? Although the USA and China can be regarded as the two major powers of the present and the future, among the Asian players, India is trying evidently to catch up with those two countries as a major power, albeit lagging perhaps one or two laps behind them. Perhaps, India needs some more time to emerge as a major world power, wielding its increasing national power to back up an international order–building capability.^1^1Perkovich, for instance, points out that ‘India cannot get other important states to comply with Indian demands ... India does have the capability to resist demands placed upon it by other countries’ (Perkovich, 2003–2004). A Japanese expert of South Asian affairs argues that India’s foreign policy objective is to become a major power in terms of having the capability to alter the international system or to be perceived as a major power (Kondo, 2012, p. 7). India aspires to be a major power as soon as possible, whereas Japan wants to remain a global power for as long as possible.

Although there are various views on how the world will look after COVID-19 as shown in *The Foreign Polic*y of 20 March 2020,^2^2Refer, for example, Allen et al. (2020). I will briefly narrate the prospects of modus operandi by the USA and China. Getting straight to the point, it would be inconceivable that either of them plays an expected role.

### The USA

In fact, after the end of World War II, the USA emerged as the victorious nation; with its incomparable national power—accounting for half of the global GDP—and extraordinary military capabilities, it led the founding of political and economic institutions such as the United Nations (UN), the World Bank, and the International Monetary Fund (IMF). Even in 1960, the USA’s GDP represented 40 per cent of the entire global GDP (Patton, 2016).

Now, in the twenty-first century, the USA remains the most influential major power. However, its proportions of GDP (World Bank) and also defence expenditure (SIPRI) are roughly one-fourth that of the world. The USA is not at all predominant, as it was in the twentieth century. Therefore, it is only barely in a position to renew the global or regional order established in the last century, let alone lead a new global order or a new regional order of the Indo-Pacific. In short, the Indo-Pacific is in the midst of a power transition.

Nevertheless, as recently as May 2014, President Obama told a crowd of cadets at West Point that the USA remains an ‘indispensable nation’ that will face down terrorism threats around the world and work to bolster key allies while avoiding costly and open-ended wars.

It seems that the USA will never be accommodative of China’s claim for greater territory and influence. A presidential nominee for the Director of the CIA, Gina Haspel testified to the Senate in May 2018:

For decades, American foreign policy towards China has been rooted in the belief that as they prospered economically they would embrace democracy, they would embrace the global rule of law. That consensus I think by all accounts has been catastrophically wrong. Today China is undertaking a comprehensive effort to supplant the United States and to undermine us.^3^3See https://www.intelligence.senate.gov/hearings/open-hearing-nomination-gina-haspel-bedirector-central-intelligence-agency

Similar stern views on China have been stated in the 2018 report of the USA–China Economic and Security Review Commission, a bipartisan advisory body of the US Congress. The views have been legislated as the Asia Reassurance Initiative Act 2018 (ARIA). ARIA has stipulated how to cope with the rise of China through the quadrilateral consultation/cooperation (Quad) framework, through Japan–USA relations and through expectations of India as the major defence partner, besides Australia. In such a way, it is possible to say that the USA has altered its traditional policy towards China.

### China

China might be classified as a possible major power in terms of regional order–building capability. Soon after Xi Jinping became the General Secretary of the Chinese Communist Party in November 2012, he put up the slogan of *the Chinese Dream,* which consisted of the two founding centenaries: China becoming a ‘moderately well-off society’ by 2021, which is the 100th anniversary of the party foundation, and in 2049 reaching the modernisation goal of becoming a fully developed nation (Kuhn, 2013).

Xi’s Chinese Dream has been accompanied by his proposal to establish a New Type of Great Power Relations between the USA and China. His view has been repeated twice to the USA president to the effect that the USA should recognise China as a great power and that the Pacific Ocean should be regarded as the two countries’ sphere of influence.

On his meeting with President Obama in June 2013 in Palm Springs, California, Xi said: ‘When I visited the United States last year, I stated that the vast Pacific Ocean has enough space for the two large countries of China and the United States’. An almost identical statement was repeated by Xi to President Trump during the latter’s visit to China in November 2017: ‘As I said to the President, the Pacific Ocean is big enough to accommodate both China and the United States’.

### After COVID-19

Given a background of the animosity between the USA and China over the regional order, in the wake of the COVID-19 outbreak, the bilateral relations have changed for the worse. Both the countries are engaged in a mutual propaganda war. Although the contours of the post-pandemic order remain to be seen, one matter seems certain: far from normalising their relationship, the USA and China are likely to become increasingly estranged and increasingly hostile (Pei, 2020).

President Trump said on 30 April 2020, without providing further details, that he has seen evidence linking the novel coronavirus to a lab in Wuhan, China. Even within the USA, Trump and presumptive Democratic presidential nominee Joe Biden have seized on convenient stratagem: each accusing the other of being a stooge for Communist China.^4^4Refer, for example, *The Washington Post* (2020). The World Health Organization’s (WHO) favourable appraisal of Chinese success stories has caused US President Trump to freeze the USA’s WHO funding.

The spokesman for China’s Foreign Ministry suggested on 12 March 2020 that the US military might have brought the coronavirus to the city of Wuhan, which has been hardest hit by the outbreak. China has never accepted the responsibility of the spread of COVID-19 or showed a willingness to disclose details about what happened in Wuhan. All data presented as statistical data related to COVID-19 in China have become suspect because of the lack of transparency.

It might be quite natural for Trump and Biden to engage in a heated debate in this year of the US presidential election. Their rhetorical statements tend to be abrasive and hyperbolic. USA–China relations seem to be the most important foreign policy issue of the November 2020 presidential election.

Besides, China would be only slightly amenable to global criticisms of its transparency, although China is showcasing itself as the alternative to the Western world in providing public goods and services to the virus-affected regions (Kondapalli, 2020).

According to Reuters’ news of 14 April 2020, China’s economic rate was forecast as 2.5 per cent, which would mark its weakest since 1976. Xi has to overcome his biggest and hardest conundrum damaging his slogan of the Chinese Dream besides the problems of Xinjiang and Tibet and so on.

It is no exaggeration to say that the mode of the USA and China^5^5According to the PEW opinion conducted in April–May 2019, people in the Asia-Pacific regard the USA more favourably than China, but Trump gets negative marks. See https://www.pewresearch.org/fact-tank/2020/02/25/people-in-asia-pacific-regard-the-u-s-more-favorably-than-china-but-trump-gets-negative-marks/ in dealing with global issues such as COVID-19 is short of altruism. If the two countries pretend to be world major powers, they must show their responsibility and lead the world towards combatting the epidemic based on the notion of *noblesse oblige*. In other words, the two countries lack sufficient national power to create or manage a new type of global order, to say nothing of supporting an Indo-Pacific regional order.

## Japan–India Relations as a Public Good

As long as the USA and China behave mainly according to self-interested notions without bothering about leading other countries towards renewing or rehabilitating existing international institutions, and as long as the two countries dare not to do something to overcome COVID-19, what should other countries or international organisations do?

I am of the view that many countries around the world are tidying up their houses and are unable to be concerned with others. Furthermore, no one knows when COVID-19 will cease to be a threat. Despite great efforts, most nations are not equipped to fight the disease solely based on their medical systems and stimulation of their weakened economies. Every country has started preparing its exit strategy. However, no one can foresee all the pitfalls waiting in the post-pandemic world.

The best time to ponder over a future framework of the Indo-Pacific is now. Some would say it is too early. However, there is a lack of leaders and leading countries to shoulder such functions. Under such circumstances, Japan and India should initiate consideration of a stable and democratic regional order. The relationship of the two countries could be formulated preferably as a public property (or good) to be shared by all countries in the Indo-Pacific (Horimoto, 2013, pp. 35–36).

In the past, Japan–USA relations have sometimes been compared to a public good serving the interests of Asia, which could have been regarded as contributing to the stabilisation of the Asian political environment. In other words, the relations are meant not for beneficial bilateral relations as a private property but for the peace, stability and development of the Indo-Pacific as a whole.

However, the Japan–USA relationship has tended to be regarded as a relation between a junior partner and a senior partner. Therefore, the discourse of Japan–USA relations as a public property has not been readily accepted in the region during the twentieth century. This characterisation of the relationship has been especially true from the perspective of India. Satu Limaye pointed out that India had written off Japan as an American surrogate in Asia during the Cold War period (Lymaye & Sato, 2006, pp. 226–227).

It is possible to say Japan's foreign policy relative to India has resembled America's India policy in the post-Cold War era (Tamari, 2013, p. 169). Japan’s policy towards India has shown a gradual evolution in the earlier 2010s. Now there is an opinion that Japan should cooperate to build connectivity infrastructure inside and outside of India and play a more active role to bridge between Modi’s India and Trump’s U.S (Ito, 2020).

It would be an opportune time for Japan and India to look to the future, particularly from the perspective of their post-COVID-19 situations. The two countries are major countries in the Indo-Pacific now. Their relations are the closest ones for renewing existing institutions and creating new mechanisms.

More specifically, strategic cooperation between Japan and India should be so oriented to create an Indo-Pacific environment in which China would find it difficult to become a hegemon and would instead be compelled to accept cooperation within a regional framework. Moreover, it must be emphasised that such a framework should be formulated in an inclusive manner to remain effective and sustainable in future decades. Japan–India relations have sufficient potential to emerge as a public property benefitting all Indo-Pacific countries and not benefitting the two countries themselves. However, the author intends to say that USA and China should not be written off in discussing the regional order of the Indo-Pacific.

## Japan’s Policy of Indo-Pacific Order: FOIP, Quad and RCEP

The regional order of the Indo-Pacific propounded by Japan consists of three strategic policies: FOIP, representing the regional order; the Quad being the political/military pillar; and the RCEP, the economic pillar, apart from its naval build-up. These policies have had the objectives of coping with the Chinese emergence in the Indo-Pacific through policies of the Belt and Road Initiative (BRI), the China–Pakistan Economic Corridor (CPEC), Asian Infrastructure Investment Bank (AIIB) and naval build-up for achieving regional dominance. Although naval power build-ups are indispensable for achieving other strategic objectives, they are minimised for brevity in this discussion.

### Free and Open Indo-Pacific

Japan has been promoting the concept of FOIP since the mid-2010s. The original expression was put forth in Prime Minister Abe’s keynote address at the Tokyo International Conference on African Development (TICAD) VI held in Kenya in August 2016: ‘What will give stability and prosperity to the world is none other than the enormous liveliness brought forth through the union of two free and open oceans and two continents’.^6^6See https://www.mofa.go.jp/afr/af2/page4e_000496.html Suffice it to say here that the TICAD has another important agenda of connectivity development.

The Ministry of Foreign Affairs (MOFA), Japan, has referred to his address as the first use of FOIP in its annual report of the Diplomatic Blue Book 2017, even though there had been plenty of similar phrases used by scholars and experts in Japan and overseas by that time.

The Japanese Government has clarified the basic concept of FOIP on its website:

Develop a free and open Indo-Pacific region as ‘international public goods’, through ensuring the rule-based international order, in a comprehensive, inclusive and transparent manner, attaching importance to ASEAN’s centrality and unity, to bring stability and prosperity for every country as well as secure peace and prosperity in the region as a whole. Japan will cooperate with any country that supports this idea. (Government of Japan, 2019)

The website carries the gist of FOIP through three headings: ‘Promotion and establishment of the rule of law, freedom of navigation, free trade…, Pursuit of economic prosperity, Commitment for peace and stability’.

After the introduction of FOIP, debates have raged over its efficacy and winning the favour of other countries concerned. Originally, the MOFA used the FOIP strategy. However, since the middle of 2018, it has replaced the strategy with vision, the FOIP vision. The term strategy carries an implication of hard political reality with a strong orientation of realising it, so, perhaps, the MOFA has come to accept the use of the softer term—the FOIP vision.^7^7See Koga (2020) for its excellent analysis.

Such changes might be more amenable to India and the ASEAN countries, as discussed later. Some similar and previous precedents exist. China’s BRI was originally the BRI strategy, subsequently changed to the BRI initiative.

### Quad

It would be necessary to take note of the relation between FOIP and the Quad, although it might be rather difficult to comprehensively define it. Applicably, FOIP signifies an overall objective in the Indo-Pacific to be implemented to be the regional order while the Quad might signify one of the capabilities to guarantee the realisation of FOIP. One Japanese security specialist on South Asian of Japan compares it recently to ‘teeth’.

In a press release issued on 12 November 2017 titled ‘Australia-India-Japan-U.S. Consultations on the Indo-Pacific’, the MOFA reported, ‘Senior officials of diplomatic authorities in Japan, Australia, India, and the USA met in Manila, the Philippines on 12 November, and discussed measures to ensure a free and open international order based on the rule of law in the Indo-Pacific’, and noted that ‘The participants affirmed their commitment to continuing discussions and deepening cooperation based on shared values and principles’ (Ministry of Foreign Affairs, 2017). Another round of four-way talks was held in June 2018. This FOIP might be termed as the second one because it was the successor of the first FOIP (Horimoto, 2017). Finally, the Quad of four countries has been upgraded to the ministerial level interaction from the secretary level in September 2019 during the UN General Assembly in New York. These Quad discussions have represented a step towards the realisation of FOIP.

There appear to be distinctive features of the recent Quad. First is Japan’s role as the main mover behind the talks. Second is India’s full-fledged participation. The USA appears to have been more of a supporter rather than a leader. Australia has also supported the Quad process, as seems only natural in light of its alliance with Japan and the USA.

An important difficulty persists about the degree of commitment to FOIP on the part of the ‘America first’ Trump administration. Under President Barack Obama, the USA was promoting the Trans-Pacific Partnership (TPP) as the mainstay of its economic policy towards Asia, paired with a ‘rebalance to Asia’ as its overall strategic policy for the region. However, Trump has taken the USA out of the TPP and has abandoned the Asian rebalancing policy altogether. T. J. Pempel of the University of California has summed up Trump’s first 12 months as a period of ‘absenteeism from Asia’ (Pempel, 2017).

When Trump made his first Asian tour in November 2017, because he had no Asian policy of his own, simply going along with Japan’s FOIP initiative was his only remaining alternative. He was compelled to agree with Abe.

Even before Trump’s visit, however, officials of the US administration had been discussing the Quad. For example, in an address on 18 October 2019, Secretary of State Rex Tillerson spoke of extending the trilateral engagement among the USA, India and Japan to include Australia. Furthermore, the National Security Advisor H. R. McMaster was said to have started frequently using the term ‘Indo-Pacific’ shortly before President Trump’s Asian tour.^8^8Refer, for example, *Japan Times*, 4 November 2017.

Then, in the National Security Strategy released by the White House in December 2017, the USA clearly expressed its wariness towards China and Russia and a desire to promote the Quad, declaring, ‘China seeks to displace the USA in the Indo-Pacific region, expand the reaches of its state-driven economic model, and reorder the region in its favour’, and stating, ‘We welcome India’s emergence as a leading global power and stronger strategic and defense partner. We will seek to increase quadrilateral cooperation with Japan, Australia, and India’.^9^9Refer, for example, White House (2017, pp. 25, 46). https://www.whitehouse.gov/articles/new-national-security-strategy-new-era/

It would be impossible to attribute the policy change entirely to the change of the US government. In a pre-Trump-era work, Sheila Smith, an American scholar on Japan, noted the importance of both China and Japan for the USA, writing, ‘The biggest challenge for U.S. policymakers will be developing a cooperative relationship with Beijing while not undermining the US’ close alliance with Tokyo’(Smith, 2015, p. 260). In this respect, the Quad framework at the diplomatic level is probably the most suitable approach for Washington at this point.

The USA has published two official policy papers: *National Security Strategy*, 2017, and *National Defense Strategy*, 2018. These two papers, in short, made China the key objective in strengthening US military forces and single it out as America’s primary strategic competitor. Terrorism is not its main concern.

John Mearsheimer, an American international political scientist, pointed out that for the USA, which has a rich history of acting as an ‘offshore balancer’, the ideal strategy for dealing with China is to leave the task of containing it almost entirely up to the countries of the region, remaining in the background to the greatest extent possible (Mearsheimer, 2014, p. 385). The USA is a supporter of FOIP and the Quad rather than a promotor.

China has opposed persistently the Quad since its inception in 2007. When such Australia–India–Japan–US moves first surfaced, the Chinese showed a strong negative reaction. They view such frameworks, whether quadrilateral or trilateral (involving the USA and two of the other three countries) as being aimed at encircling them (Garver & Wang, 2010).

China has reacted to the upgrading of the Quad to the ministerial level by stating ‘no need to overreact’ (Zhao, 2019), although the Quad fundamentally represents a North Atlantic Treaty Organization (NATO)–like group in Asia. Their criticism and objections against the 2007 Quad and the 2017 Quad have been fundamentally equivalent in tone (Horimoto, 2020).

One Chinese expert wrote that the four-way talks were aimed at containing China and warned that they would hinder regional development (Lian, 2017). Another declared that the FOIP strategy, aimed at blocking the BRI, was doomed to fail (Zhao, 2019).

In a press conference on 13 November 2017, Geng Shuang, Deputy Director of the Chinese Foreign Ministry’s Information Department, expressed concern, noting that such multilateral initiatives should promote cooperation among the countries concerned and not be turned into exclusionary frameworks. It would be rather natural for China to object against the Quad, because it might become a stumbling block for the region to realise a *Pax Sinica* dream.

## India’s Indo-Pacific Policy

When discussing the Indo-Pacific region, Japan–India relations are indispensable. From a bird’s-eye view, Japan–India relations have got on the right track in the early part of the twenty-first century. The major factor cementing the two countries has been a measure to cope with the rise of China, aside from bilateral economic and security cooperation, initially in Asia and then later expanded to the Indo-Pacific.

### Realistic Foreign Policy

The two countries have similar perceptions about the rise of China, but a subtle difference remained in the level of measures to be taken. In the strategic thoughts of India, there might be realistic thinking, as Kanwal Sibal, ex-Foreign Secretary, said:

Japan’s economic stakes in China are huge; our own political and economic stakes in China are high. Neither Japan nor India seeks a confrontation with China, but both have a responsibility to build lines of defense against any disruptive exercise of power by a rising China. (Sibal, 2014)

In a similar vein, Sandy Gordon of Australia National University opined that the Modi government might attempt to play both ends against the middle among China, the USA and Japan (Gordon, 2014).

Looking specifically at the Indo-Pacific policy in the Bharatiya Janata Party’s (BJP) Manifestos, the 2014 Manifesto included no words about it. However, the 2019 Manifesto listed several points in The Foreign Policy section, which mentions the UN, G20, BRICS, SCO, the Commonwealth and interactions such as Russia–India–China (RIC) and Japan–America–India (JAI) and Neighbourhood First policy through BIMSTEC. Furthermore, it proceeds to ‘Act East Policy, cooperation with ASEAN and ensuring an open, inclusive, prosperous and secure Indo-Pacific will be pursued vigorously’. These policies sound comprehensive in their nature and scope.

### The Indo-Pacific: India’s Main Diplomatic Battlefield

Presently, it can be safely assumed that India has a clear strategic objective to become a major power of the world. Presently, the USA is the only superpower. China chases it, as does India, although one or two lengths behind China. No other country in the world appears likely to aspire to be a superpower from simply a major power.

If summarised, the key point of Indian diplomacy as pursued by the Modi government can be categorised as having three levels: global, regional (Indo-Pacific) and local (South Asia) (Horimoto, 2017). At the global level, India cooperates with China and Russia to foster the creation of a multipolar international order as the early stages of its quest for major-power status. As a parallel endeavour, it also engages in efforts to boost its national prosperity and military strength.

At the regional (Indo-Pacific) level, however, it endeavours to develop a larger presence and evolve into a maritime power and advance its Act East policies in the political and economic spheres through cooperation with Japan, the USA and Australia. Policies at the local level (South Asia) will comprise a subset of regional-level efforts and an intention to secure its hegemonic position. At this level, India is currently concentrating its efforts to counter China (Horimoto, 2019).

New Delhi’s main troubles are with antagonists at the regional level (particularly China) and the local level (particularly Pakistan). Now that China and Pakistan are moving even closer together with the development of the CPEC, India may look at the pursuit of the FOIP strategy and the Quad as a realistic option for hedging against China while avoiding the instigation of excessive friction.

At the regional level (Asia, Western Pacific, the Middle East, Africa and the Indian Ocean), India is striving to attain a dominant position and display its relative presence through joining hands with the USA, Japan and others while facing China. At the moment, the regional level is India’s main battlefield in terms of strategic implications.

The expansion and enhancement of the Indian Navy’s activity have been pursued based on a ‘diplomatic’ and ‘benign’ role in line with the role of ‘security provider’ that the USA expects of India. In the 2015 *Maritime Security Strategy*, India supports freedom of navigation. India is also endeavouring to build up its capability as a ‘net security provider’. Specifically, it has supported capacity building for Sri Lanka, the Maldives, Seychelles and Mauritius. India has also sought security cooperation from member countries of the ASEAN, the USA and Japan against the backdrop of the South China Sea issue (Izuyama, 2017).

### India’s FOIIP

India has shown its traditional wariness in the case of FOIP and the Quad (Horimoto, 2019), yet to commit itself fully to the FOIP strategy and the Quad. As noted in the 2017 Diplomatic Bluebook of the MOFA:

During Prime Minister Narendra] Modi of India’s visit to Japan in November 2016, the two leaders shared the view to take the initiative for the stability and prosperity of the Indo-Pacific region by enhancing the synergy between Japan’s ‘Free and Open Indo-Pacific Strategy’ and India’s ‘Act East Policy’ through collaboration.^10^10Refer, for example, MOFA, Diplomatic Blue Book, 2017, p. 27.

But New Delhi has not given its full approval to FOIP and the Quad. In his keynote address at the 2018 Shangri-La Dialogue on 1 June 2018, Prime Minister Modi used the term ‘inclusive’ four times, stressing that the Indo-Pacific envisaged by India must be a free, open, *inclusive* region—‘FOIIP’ rather than ‘FOIP’. Prime Minister Abe also said in his January 2018 policy speech to the National Diet (Parliament) that Japan would ‘work with China’ based on *the overall direction of the FOIP strategy*.^11^11The Ministry of Foreign Affairs has clarified the basic concept of FOIP in its website as ‘Develop a free and open Indo-Pacific region as “international public goods”, through ensuring the rule-based international order, in a comprehensive, inclusive and transparent manner, attaching importance to ASEAN’s centrality and unity, in order to bring stability and prosperity for every country as well as secure peace and prosperity in the region as a whole. Japan will cooperate with any country that supports this idea’. (https://www.mofa.go.jp/policy/page25e_000278.html) His inclusiveness appears to be no more than diplomatic rhetoric.

In other words, Modi’s references to inclusiveness rhetorically represent the inclusion of China. For New Delhi, relations with Beijing could be regarded as a top foreign policy priority. To maintain a stable bilateral relationship, India has been both engaging China and hedging against it (Horimoto, 2018a). Or, put in another way, at the moment, India has the necessity of managing its relations with China, as pointed out by many of India’s China specialists. I add hastily, there emerge various hard-line opinions *vis-à-vis* China, which deploys arbitrary and selfish policy orientations (Rajagopalan, 2020). And as a corollary of it, an India expert advocates closer relation with the USA (Raja Mohan, 2020).

New Delhi can use the Quad as a hedge against Beijing, taking the Chinese criticism of the four-way framework (the Quad) as deriving from the bureaucratic rather than the political level. While continuing to be a regular member of the Shanghai Cooperation Organization and participate in the BRICS summit (an annual gathering of the leaders of Brazil, Russia, India, China and South Africa), India might look to be accepting to upgrade the Quad to the foreign-ministerial or prime-ministerial level. From 2002 through 2019, India has held the RIC foreign ministers’ meeting among Russia, India and China on 16 occasions.

India has been hesitant and wary to upgrade the participants to the ministerial level (Horimoto, 2018b). However, India took part finally in the Quad meeting of foreign ministers in New York held in September 2019.^12^12Refer, for example, *The Economic Times* (2019). Perhaps, the modification of the FOIP vision from the FOIP strategy along with its basic orientation brought about by the modification might have been conducive for India’s upgrading.

New Delhi’s Indo-Pacific strategy is informed by the idea of balancing China and Russia against Japan and the USA. It may be seen as an expression of ‘strategic autonomy’, a term that has been used widely within India as an expression of the country’s new foreign policy stance since the start of the current decade.

### From Act East to Act Indo-Pacific

During the Modi government administration, which has been governing since 2014, one can notice various new policy initiatives in the Indo-Pacific. These have been the Act East policy from the Look East policy as declared by the Foreign Minister Sushma Swaraj on 26 August 2014. In March 2015, Prime Minister Modi unveiled India’s strategic vision for the Indian Ocean Security and Growth for All in the Region (SAGAR) during his tour of Seychelles, Mauritius and Sri Lanka. SAGAR intended to differentiate India’s leadership from other regionally active major powers and also to reassure littoral countries as India’s maritime influence grows (Estrada, 2020). It should be noted that for Japan also the Indian Ocean carries strategic importance (Nagao, 2019).

**Figure 1.**
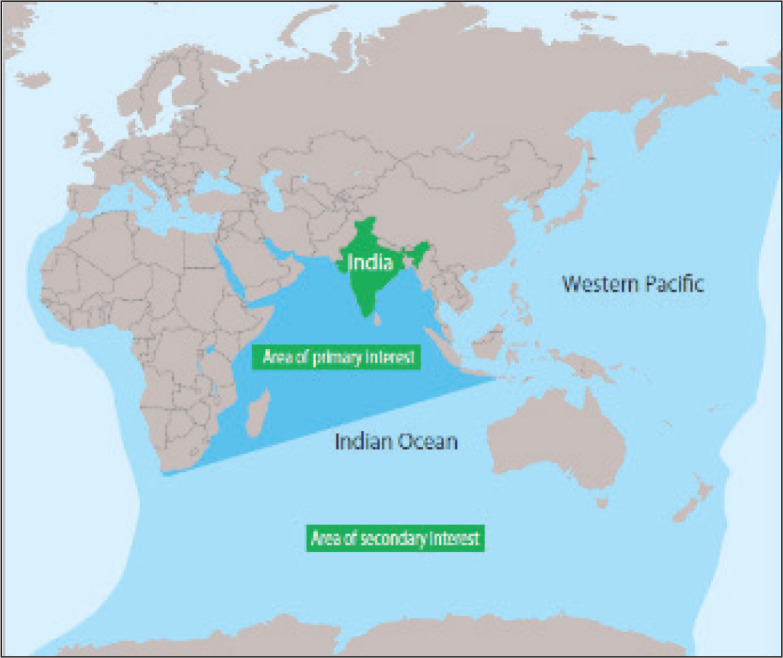
Maritime Areas of Interest to India

**Source:** Khurrana (2017).

SAGAR has been followed by the Indian Navy’s clear-cut image of Maritime Areas of Interest to India in the Indo-Pacific shown by Gurpreet S. Khurana’s image (Figure 1). An almost identical drawing is presented in the official document of the Indian Navy (Indian Navy, *Ensuring Secure Seas: Indian Maritime Security Strategy*, 2015).^13^13See https://www.indiannavy.nic.in/content/indian-maritime-security-strategy-2015

Prime Minister Modi urged at the Meeting of Heads of Indian Missions in February 2015 to position India in a leading role rather than as a balancing power (later, Foreign Secretary Jaishankar stated ‘a leading power’ at his IISS-Fullerton Lecture, Singapore, in July 2015). Chronology of this kind would lead to a lengthy exposition, but it can be summarised by referring to a significant initiative.

In viewing this way, India’s Indo-Pacific policy has been transformed significantly, covering the whole gamut of the ocean. It is quite natural for Prabir De to characterise the policy change as *Act East to Act Indo-Pacific* (De, 2020a, 2020b).

## Perspectives for Multilateralism

At the moment, COVID-19 has been raging throughout the world and infecting 3.6 million persons and killing more than 250,000. Yet, the disease is spreading without a sign of halting. We cannot rule out the second or the third waves of COVID-19 in regions where strict measures have somehow been relaxed. Perhaps, COVID-19 will have strong effects on the course of history when considering its various developments of situations.

If the necessary steps are not taken, international anarchy could intensify. As Rudd pointed out, ‘there is no “system manager,” to borrow Joseph Nye’s phrase, to keep the international system in functioning order. It may not yet be Cold War 2.0, but it is starting to look like the Cold War 1.5’ (Rudd, 2020).

Now, it is the time to ponder over an exit strategy—the sooner the better. The strategy should not remain only to resurrect and upgrade medical systems and economic activities. President Trump on 6 May 2020 called COVID-19 ‘worse than Pearl Harbor and the 9/11 attacks’. However, his characterisation is limited to the USA only. True to form, he thinks merely in terms of the USA, not of the globe as a whole. I would like to emphasise that there should be wider perspectives of a set of basic approaches and measures and means and ends. The set should include multilateralism and global or regional order as the first step.

The USA and China are not in a position to take the lead, fortunately or unfortunately. The USA holds its presidential election in November 2020 and is not at all in the mood to think globally. Furthermore, the USA is unlike itself soon after World War II, because of the relative decline of its national power and status. Joseph Nye argued ‘even if the US remains the largest power, it cannot achieve many of its international goals acting alone” because of the information revolution and globalization’. The Trump administration has focused almost entirely on great power competition, particularly with China (Nye, 2020).

China, which aspires to gain an international order-building capability, has not yet reached the stage in terms of national power. Moreover, China’s efforts to pretend to be ignorant of the origin of the virus neglect its duty of transparency to the world. Like the USA, China is also unfit for the role. Although some argue that Trump and Xi must cooperate against COVID-19, it now seems like wishful thinking (Bollyky & Kupchan, 2020).

Haas has pointed out, ideally, that the crisis would bring a renewed commitment to building a more robust international order, much as the cataclysm of World War II led to arrangements that promoted peace, prosperity and democracy for nearly three-quarters of a century…and no other country, not China or anyone else, has both the desire and the ability to fill the void the USA has created (Haass, 2020).

What we need now in the Indo-Pacific as the starting area is an effort for which the keyword should be *multilateralism.* Such ideas should be expanded to other regions also. There appear several opinions in support of or that go by multilateralism.^14^14Refer, for example, Mattoo and Nalikar (2020), Rouse and Triggs (2020) and Oanh (2020). For instance, Mattoo and Nalikar propose to resuscitate multilateralism with India’s help (Mattoo & Nalikar, 2020). Rouse and Triggs also stress the importance of making use of G20 (Rouse & Triggs, 2020). It is a wonderful insight, though there remains a question of who will take the lead.

This author would like to stress the importance of a stepwise approach. An idea of multilateralism is an excellent strategy; however, when it touches upon a concrete and actual measure, many countries and with their individual agendas and interests are concerned. Therefore, a gambit of a concrete measure should undertake COVID-19 and related issues which requires urgent attention. In this sense, COVID-19 and related issues would be the *raison d’etre* accompanied by an urgency to tackle with. Modi has said India–Japan partnership can help develop new solutions for the post-COVID-19 world, on 10 April 2020.

### Japan and India with a New Indo-Pacific Vision

Under such situations, possible players are expected to be Japan and India: not separately but in union to create an atmosphere conducive to employment by the two countries. India has been known to the world through its use of normative thinking, such as non-alignment by Nehru, and Narendra Modi, after he assumed the office,^15^15Refer, for example Nidhi (2020). enhanced India’s role in international affairs as a *vishwaguru* (world guru) (Hall, 2017). The two countries should make use of their close relations for the public good of the Indo-Pacific.

It would be true to say the bilateral relations between Japan and India have been furthered in recent years (Jain, 2019; Roy, 2020). The two countries should make use of their close relations for the public good of the Indo-Pacific. The prime ministers of the two countries are encountering the severest domestic conditions *vis-à-vis* COVID-19, with insufficient time to embark on an international effort. Their undertaking would enhance their credibility at home and abroad, particularly for Abe, whose approval rate is dwindling (Kesavan, 2020).

## Conclusions and the Way Forward

Under the current global configurations of powers and situations, in this article, I would propose three points to be emphasised for the future. First, countries of the Indo-Pacific should strive for sharing the concept of multilateralism as the basic regional principle. Consequently, no country should dominate the region for its interests.

Second, whether FOIP or FOIIP, democracy should be added as one basic tenet (Chellaney, 2020). The new abbreviation is FOID (Free, Open, Inclusive, Democratic Indo-Pacific). Multilateralism cannot function properly without democratic orientation. G20 and the East Asian Summit are possible gatherings and platforms for proceeding with multilateralism.

Third, how to keep the ASEAN’s centrality in the architecture of the Indo-Pacific (Basu, 2020). Active involvements of the ASEAN in such multilateral initiatives are sine quo non. In this connection, India’s involvement in the RCEP is essential, although India has its compulsion to stay out of the RCEP (Horimoto, 2020). Kishore Mabhubani compared the last-minute announcement to Britain’s Brexit decision and predicted that ‘India will come to realize that “Look East” and “Act East” policies will mean absolutely nothing if it does not join RCEP’ (Mabhuban, 2019).

Without India, Japan’s influence in the RCEP would be significantly reduced. Although Beijing has publicly called on New Delhi to reconsider, one can easily imagine that the Chinese are privately pleased with the latest turn of events.

FOID aims at achieving a stable regional order in the Indo-Pacific. Such an order would cover greater cooperation to monitor outbreaks of infectious diseases and deal with their consequences, as well as a greater willingness to address climate change, set rules for cyberspace, assist forced migrants and tackle the proliferation of terrorism, to say the least of economic and trade matters.

As a corollary to FOID, the Quad should be regarded as expansive and not exclusive. It should be directed towards a Quad-Plus (Rajagopalan, 2020a). It is interesting to view the recent move by the Indian foreign secretary between 20 March 2020 and 15 May 2020, as if he had such an expansive Quad in mind. He talked about COVID-19 with countries in the Indo-Pacific such as the USA, Australia, Japan, the Republic of Korea, New Zealand and Vietnam.^16^16Refer, for example, *The Economic Times* (2020). Probably, the talks have been done in conjunction with the 18–19 May 2020 talks of the World Health Assembly, whose main agenda was expected to be an inquiry into COVID-19 and Taiwan’s observer participation.

We are at the crossroads of a global predicament. It is imperative to turn the tide of the COVID-19 situation and find a lifeline to a safe and prosperous future in the Indo-Pacific so that we can find a silver lining for our future (22nd May 2020).
